# Lamb behaviours in the first three hours postnatal in the Kivircik breed: associations with growth performance and influencing factors

**DOI:** 10.1007/s11250-026-05029-2

**Published:** 2026-04-11

**Authors:** Bulent Ekiz, Hulya Yalcintan, Omur Kocak, P. Dilara Kecici

**Affiliations:** Department of Animal Breeding and Husbandry, Faculty of Veterinary Medicine, Istanbul University-Cerrahpasa, Buyukcekmece Campus, Buyukcekmece, Istanbul, 34500 Turkey

**Keywords:** Postnatal lamb behaviours, Lamb growth, Maternal care

## Abstract

The aim of this study was to determine the relationship between lamb and ewe behaviours in the first three hours after birth, and lamb growth rate up to postnatal day 60. The effects of lamb sex, birth type, and dam parity on lamb behaviours in the first three hours postnatal were also investigated. Thirty-six Kivircik ewes and their 55 lambs were used in the study. According to the stepwise regression analysis, Model 2, which included ewe and lamb behaviours as well as lamb birth weight as predictors, resulted in higher R^2^ and lower root mean square error (RMSE) compared with Model 1, where only ewe and lamb behaviours were explanatory variables. Grooming by the dam during the first 30 min and standing duration during the first 180 min were the most important behavioural predictors of lamb growth. In conclusion, the growth rates of Kivircik lambs over the first 60 days postnatally could be predicted with moderate accuracy (with R^2^ values ranging from 0.492 to 0.705) by considering birth weight alongside lamb behaviours observed during the initial three hours postnatally as explanatory variables.

## Introduction

The profitability of sheep farming enterprises aimed at meat production is largely associated with the high survival and growth rate of lambs (Bruce et al. 2021). The behaviours exhibited by the ewe and lamb postpartum facilitate the establishment of a bond between the mother and her offspring. The bond formed between the ewe and her lamb can have an impact on various characteristics of the lamb’s long-term physiological and behavioural development, as well as growth performance (Karaca et al. 2023; Rooke et al. 2017).

It has been reported that the genotype of both the ewe and the lamb, along with various factors such as birth type, dystocia, previous maternal experiences, nutritional status during late pregnancy of ewes, the sex and birth weight of the lamb, and environmental conditions in the lambing pen, can influence the bond established between the lamb and its dam, as well as postnatal lamb behaviours (Dwyer 2008; Karaca et al. 2023). Understanding the effects of these factors on lamb behaviours could reduce lamb mortality shaped by behavioural problems by improving physical conditions during or after lambing or by implementing methods to strengthen the relationship between the ewe and her offspring in the early stages (Nowak 1996).

Previous research has investigated postnatal lamb-ewe behavioural interactions across different sheep breeds and has identified potential risk factors for lamb mortality (Cloete et al. 2021; Mialon et al. 2023; Odevci et al. 2021). However, studies specifically addressing the association between postnatal lamb behaviours and early growth remain limited (Everett-Hincks et al. 2005; Madani et al. 2013; Mialon et al. 2023). Furthermore, the strength of the postnatal bond between an ewe and her lamb has been reported to vary substantially among sheep breeds, with indigenous and hill breeds generally exhibiting stronger maternal-offspring bonds than intensively selected or lowland breeds (Dwyer and Lawrence 2005; Fonseca et al. 2024). Kivircik is a medium-sized indigenous breed originating from the Thrace region of Türkiye. The meat of Kivircik lambs is widely regarded as the highest-quality lamb meat produced in the country and therefore often commands a premium price (Ekiz et al. 2024). To the best of our knowledge, no studies have investigated the relationship between postnatal ewe and lamb behaviours and subsequent growth rates in the Kivircik breed. In the current study, we hypothesised that behavioural responses exhibited by lambs during the first three hours after birth, together with the maternal grooming, would be associated with preweaning growth rate. This study aims to determine whether lamb behaviours exhibited during the first three hours after birth, along with the grooming duration provided by ewes, influence lamb growth rate up to 60 days of age, and to evaluate the effects of lamb sex, birth type, and parity of dam on lamb behaviours observed during this early postnatal period.

## Materials and methods

### Animals and management

The research was conducted in the experimental sheep farm of Istanbul University Faculty of Veterinary Medicine. The procedures of the study comply with the criteria specified in the EU Directive for animal experiments (European Union 2010), the ARRIVE guidelines (Kilkenny et al. 2010), and the national regulations on the protection of animals used for experimental and other scientific purposes (Anonymous 2011). In the study, postnatal 3-hour behavioural data and growth data up to 60 days (15th day, 45th day, 60th day body weights and average daily gain in the first 60 days) of 55 pure Kivircik breed lambs were recorded. The research animals were the lambs of 36 pure Kivircik breed ewes. Nineteen of these ewes gave birth to twins, and 17 gave birth to singletons. The ewes were not given concentrated feed in the first 3.5 months of pregnancy. During this period the ewes were grazed in the pasture during the day, and good quality alfalfa hay (89.3% dry matter, 14.8% crude protein, and 7.51 MJ metabolisable energy /kg dry matter) was given in the evenings. In the last 6 weeks of pregnancy, the ewes were given 250 g/day/sheep commercial concentrate feed (88.1% dry matter, 16.7% crude protein, and 10.72 MJ metabolizable energy /kg dry matter) in addition to dry alfalfa hay.

In order to separate the ewes approaching birth from the main herd, a compartment with dimensions of 5.0 m × 9.0 m and a capacity of approximately 18–20 sheep was built inside the sheepfold. Straw was laid on the floor of this compartment as a bedding material. In addition, eight individual lambing pens of 1.5 m × 2.0 m were built in the sheepfold to separate the ewes that were close to giving birth from other ewes and to record postnatal behaviours.

Lactating ewes were offered 500 g/day of concentrate feed along with dry alfalfa hay. Lambs remained with their dams during the first month after birth. Thereafter, two flocks consisting of the lambs and their dams were formed, and the lambs were allowed to suckle for 2 h twice daily. Until the lambs reached 60 days of age, they were kept in an indoor pen and were provided ad libitum access to dry alfalfa hay and a growing concentrate (89.2% dry matter, 17.67% crude protein, and 11.89 MJ metabolisable energy per kg dry matter) in addition to maternal milk. Within the scope of the study, lamb weights were also recorded when the lambs reached an average age of 15, 45 and 60 days.

### Recording of lamb and dam behaviours

Cameras (Evertech 16-channel digital field switcher-Unit H, 15/F., Mai Luen Industrial Building, Hong Kong) were placed in individual lambing pens to continuously record postnatal ewe and lamb behaviours. One day before the expected start of parturition, eight ewes with advanced pregnancy were placed in individual lambing pens (1.5 m × 2.0 m area each). Good quality alfalfa hay and clean water were always available to the ewes in individual lambing pens. Behaviours of ewes and lambs were recorded in individual lambing pens for at least three hours after birth. The ewes, whose postnatal 3-hour behaviour was recorded via cameras, were removed from the individual lambing pens together with their lambs. Before a new ewe was placed in the individual lambing pen, plenty of clean bedding was spread on the floor each time. In addition, in order to avoid possible confusion when observing the camera recordings, the number of each ewe placed in the individual lambing pen was written on its back with spray paint (Akçalı spray paint, Esenyurt, Istanbul, Türkiye). All ewes in the study gave birth to their lambs and cared for their lambs without help of keepers. So, there was not any interference of the keepers with the ewes and lambs during the three hours postnatal. While the lambs were being removed from the lambing pen, they were numbered with ear tags, and their birth weight, dam number, birth type (single/twin) and sex were recorded.

### Observation of lamb and dam behaviours

In order to obtain data for lamb and mother behaviours during the postnatal three hours, behavioural images recorded in individual birthing chambers were monitored by two researchers. While monitoring the behavioural images, the postnatal latency of shakes head, to knees, attempt to stand, stands, to udder, suck attempt, and successful suck behaviours were recorded according to reports by Dwyer and Lawrence (1999), and Ekiz et al. (2024). Descriptions of lamb behaviours are presented in Table 1.

**Table 1:** Descriptions^**a**^ of behavioural traits investigated in the study

Behaviour	Description
Shakes head	Lamb raises and shakes head
To knees	Lamb on chest, pushes up on knees, supporting part of body off the ground
Attempts to stand	Lamb on knees, supports part of its weight on at least 1 foot
Stands	Lamb supports itself on all 4 feet for at least 5 s
To udder	Lamb in parallel inverse position with head nudging ewe in udder region
Suck attempt	Lamb in parallel inverse position, head beneath ewe in udder region, prevented from sucking by ewe movement or leaves udder region within 5 s
Successful suck	Lamb has teat in its mouth, in correct position, appears to be sucking for at least 5

^**a**^Behaviour descriptions are quoted from the articles by Dwyer and Lawrence (1999) and Ekiz et al. (2024)

In the study, the time spent by the lambs for suckling, standing, and lying, as well as the time received for grooming by their mothers (grooming by the dam: licking and nibbling movements of dam directed towards the lamb) were recorded in minutes for 30-minute periods during the postnatal period.

### Data editing procedures and statistical analyses

Using the behavioural data determined for 30-minute periods, the cumulative times (min) spent by the lambs at the 30th, 60th, 90th, 120th, 150th and 180th minutes for sucking, standing, lying, and also grooming by dam behaviours were calculated. The individual 15th, 45th, and 60th day weights of the lambs were determined by applying interpolation and extrapolation with the periodic daily body weight increases calculated according to the most recent weighing. Average daily gain (ADG) was calculated using birth and 60th day weights.

Before the statistical analysis, the Shapiro-Wilk test, Quantile-Quantile (Q-Q) plot of residuals, and residual plots were used to check the normality of behavioural data using the Jamovi 2.3.21 program. Since behavioural data did not exhibit normal distribution, logarithmic transformation was applied. General Linear Model (GLM) analysis was applied to logarithmically transformed data to determine the significance of the effect of lamb sex, birth type, and parity of dam on the behavioural characteristics investigated in the study. In the model used, lamb sex, birth type, and parity of dam were included as fixed effects. The same model was used in the statistical analysis of lamb growth data.

Stepwise regression analysis was applied to examine the relationship between lamb behaviour in the postnatal 3 h and the early growth of the lambs. Two different models were adapted for this purpose. In Model 1, the log-transformed data for the behavioural characteristics were used as predictors. In Model 2, birth weight was included as a predictor in addition to the predictors in Model 1. In the stepwise regression analyses, F values of 3.84 and 2.71 were used as the criterion for entry and removal of a predictor into the model. In order to evaluate the precision of the models, coefficient of determination (R^2^), root mean square error (RMSE) and significance of model were used (Eyduran 2016). Models with smallest RMSE and the highest R^2^ were considered the best models explaining the variance for lamb growth traits. Multicollinearity was evaluated by using the variance inflation factors (VIF) and A VIF value lower than 3 was considered an indicator of no multicollinearity (Khan et al. 2020). In the study, Java-based statistical processor (JASP) 0.17.2.1 software was utilised for GLM and stepwise regression analyses.

## Results

### Effects of sex and birth type of lamb and parity of dam on early postnatal behaviours and lamb growth

The effects of lamb sex, birth type, and dam parity on the latency of shakes head, to knees, attempt to stand, stand, to udder, suck attempt, and successful suck behaviours were presented in Table 2. The effects of lamb sex and birth type on the latency of these traits were not significant (*P* > 0.05). On the other hand, significant effects of dam parity on the latencies of shake head and successful suck behaviours were determined (*P* < 0.05). Lambs from second parity dams exhibited both behaviours at the latest. The lambs of fourth parity dams had sucked successfully at an earlier time compared to those of second parity dams.

**Table 2:** Effects of sex, birth type and parity of dam on latencies (min) of postnatal lamb behaviours

Factors	Data type	Shakes head		To knees		Attempt to stand		Stands		To udder		Suck attempt		Successful suck	
		Mean	SE	Mean	SE	Mean	SE	Mean	SE	Mean	SE	Mean	SE	Mean	SE
Sex															
Male	Raw	1.33	0.29	4.92	0.58	11.33	2.10	28.72	4.26	39.75	4.90	43.83	5.05	49.60	5.55
Female	Raw	1.05	0.37	4.62	0.76	8.36	2.76	22.98	5.58	33.20	6.42	36.30	6.62	48.96	7.27
Male	Log^†^	0.28	0.04	0.71	0.05	0.97	0.05	1.35	0.06	1.54	0.05	1.59	0.04	1.64	0.06
Female	Log^†^	0.23	0.06	0.70	0.06	0.93	0.07	1.27	0.08	1.50	0.06	1.55	0.06	1.64	0.04
Birth type															
Single	Raw	1.16	0.36	4.63	0.73	9.93	2.66	25.42	5.39	32.34	6.20	35.02	6.39	41.94	7.12
Twin	Raw	1.22	0.34	4.91	0.69	9.76	2.52	26.28	5.10	40.60	5.87	45.11	6.05	56.63	6.65
Single	Log^†^	0.25	0.05	0.68	0.06	0.90	0.07	1.28	0.08	1.47	0.06	1.51	0.06	1.58	0.06
Twin	Log^†^	0.26	0.05	0.74	0.06	0.99	0.07	1.34	0.08	1.57	0.06	1.62	0.05	1.70	0.05
Parity of dam															
1	Raw	1.33	0.73	5.72	1.48	8.00	5.37	19.42	10.87	36.10	12.50	38.18	12.89	60.04	14.16
2	Raw	2.03	0.39	6.17	0.79	11.94	2.87	33.35	5.81	49.10	6.68	55.68	6.89	64.23	7.56
3	Raw	0.54	0.54	3.47	1.01	11.79	3.68	25.44	7.46	32.36	8.59	36.44	8.85	40.22	9.72
4	Raw	0.86	0.33	3.70	0.67	7.64	2.43	25.19	4.92	28.32	5.65	29.95	5.83	32.64	6.40
1	Log^†^	0.22 ^**ab**^	0.11	0.80	0.12	0.98	0.14	1.20	0.16	1.56	0.12	1.60	0.11	1.74 ^**ab**^	0.11
2	Log^†^	0.41^**a**^	0.06	0.79	0.06	0.99	0.07	1.38	0.09	1.61	0.06	1.67	0.06	1.73 ^**a**^	0.06
3	Log^†^	0.17 ^**b**^	0.08	0.61	0.08	0.95	0.10	1.32	0.11	1.47	0.08	1.53	0.08	1.58 ^**ab**^	0.08
4	Log^†^	0.20 ^**ab**^	0.05	0.61	0.05	0.86	0.06	1.34	0.06	1.44	0.05	1.47	0.05	1.52 ^**b**^	0.05
P-Value^‡^															
Sex		0.426		0.891		0.619		0.429		0.528		0.510		0.998	
Birth type		0.907		0.437		0.370		0.538		0.237		0.156		0.111	
Parity of dam		0.037		0.094		0.582		0.808		0.169		0.073		0.029	

^†^Log transformed values

^‡^P-values from analysis of log transformed data

^a, b^: For a factor, the difference between mean values that do not have a common letter in the same column is significant (P<0.05)

Table 3 shows the cumulative sucking, standing, and lying durations of the lambs and the time they were groomed by their dams during the 3-h postnatal period. The sucking, standing, and lying durations of lambs were approximately 21, 100, and 59 min, respectively, during the 180 postnatal minutes. The effects of lamb sex, birth type, and dam parity on the cumulative suckling, standing, and lying durations of lambs were not significant. (*P* > 0.05). The duration of grooming by the dam was longer in single lambs than in twin lambs (*P* < 0.05).

**Table 3:** Effects of sex, birth type and parity of dam on durations (min) of sucking, standing, lying, and grooming by the dam during the first 180 min of the postnatal period

Factors	Data type	Sucking		Standing		Lying		Grooming by the dam	
		Mean	SE	Mean	SE	Mean	SE	Mean	SE
Sex									
Male	Raw	23.17	2.27	93.17	5.88	63.65	6.68	62.77	4.10
Female	Raw	19.50	2.97	106.79	7.71	53.72	8.75	57.16	3.13
Male	Log^†^	1.31	0.06	1.92	0.06	1.73	0.05	1.72	0.03
Female	Log^†^	1.20	0.07	2.03	0.08	1.71	0.07	1.78	0.04
Birth type									
Single	Raw	21.89	2.87	98.20	7.44	59.91	8.45	79.11	3.96
Twin	Raw	20.79	2.72	101.76	7.05	57.46	8.00	40.82	3.75
Single	Log^†^	1.29	0.07	1.99	0.08	1.71	0.07	1.90	0.04
Twin	Log^†^	1.21	0.07	1.96	0.07	1.73	0.06	1.60	0.04
Parity of dam									
1	Raw	14.56	5.79	93.09	15.01	72.35	17.04	58.48	7.99
2	Raw	24.01	3.09	93.31	8.02	62.68	9.10	58.74	4.27
3	Raw	25.51	3.97	109.44	10.31	45.06	11.70	62.17	5.49
4	Raw	21.27	2.62	104.07	6.79	54.65	7.71	60.48	3.61
1	Log^†^	1.02	0.14	1.96	0.15	1.88	0.13	1.76	0.08
2	Log^†^	1.29	0.08	1.86	0.08	1.72	0.07	1.73	0.04
3	Log^†^	1.37	0.10	2.04	0.11	1.61	0.09	1.74	0.05
4	Log^†^	1.33	0.06	2.03	0.07	1.68	0.06	1.76	0.03
P-Value^‡^									
Sex		0.200		0.239		0.725		0.144	
Birth type		0.404		0.769		0.759		< 0.001	
Parity of dam		0.217		0.381		0.422		0.924	

^†^Log transformed values

^‡^P-values from analysis of log transformed data

The effects of sex, birth type and parity of dam on early growths of lambs were presented in Table 4. Effects of lamb sex and dam parity on weights of lambs at 15-day, 45-day, 60-day, and ADG were not significant (*P* > 0.05). However, lamb birth type significantly influenced lamb growth rate and ADG was higher in singleton lambs (244 g) than their twin counterparts (190 g).

**Table 4:** Effects of sex, birth type and parity of dam on weights of lambs at birth, 15-day (W-15), 45-day (W-45), 60-day (W-60), and average daily gain (ADG)

Factors		Birth Weight, kg		W-15, kg		W-45, kg		W-60, kg		ADG, g		
	*n*	Mean	SE	Mean	SE	Mean	SE	Mean	SE	Mean	SE	
Sex												
Male	38	4.02	0.10	7.49	0.19	14.02	0.40	17.96	0.54	222.07	7.98	
Female	17	3.87	0.13	7.40	0.25	13.43	0.52	17.10	0.71	212.42	10.46	
Birth type												
Single	17	4.45	0.12	8.29	0.24	15.43	0.50	19.55	0.69	243.99	10.09	
Twin	38	3.44	0.12	6.61	0.22	14.02	0.40	15.51	0.65	190.50	9.56	
Parity of dam												
1	4	3.61^**b**^	0.24	6.83	0.48	12.99	1.01	16.48	1.38	208.45	20.36	
2	14	3.78^**b**^	0.13	7.30	0.26	13.66	0.54	17.32	0.74	219.41	10.88	
3	8	4.12^**ab**^	0.17	7.75	0.33	13.57	0.70	17.45	0.95	210.05	13.98	
4	29	4.28^**a**^	0.11	7.90	0.22	14.67	0.46	18.88	0.63	231.06	9.21	
P-Value												
Sex		0.287		0.741		0.326		0.290		0.421		
Birth type		< 0.001		< 0.001		< 0.001		< 0.001		< 0.001		
Parity of dam		0.013		0.125		0.282		0.238		0.545		

^**a, b**^: The differences between the least squares mean values that do not have a common letter in the same column are significant (*P* < 0.05)

### Prediction of lamb growth using behaviours in the first three hours postnatal

Results for stepwise regression analyses regarding the relationships of postnatal behaviours in the first three hours with lamb weights and ADG were given in Tables 5 and 6. In terms of lamb weights on the 15th (W-15), 45th (W-45), and 60th (W-60) days and average daily gain (ADG), stepwise regression analysis using log transformed values of lamb behaviours as predictors (Model 1) resulted in lower R^2^ and higher RMSE values than the analysis that also included birth weight (Model 2). Grooming by the dam during first 30 min (Grooming-30 min) explained alone 37.3% of the variance in W-15 (*P* < 0.001; Model 1). On the other hand, the best model for prediction of W-15 included birth weight, Grooming-30 min, stand duration during first 180 min (Stand-180 min) and latency to udder (*P* < 0.001; Model 2). This model explained 70.5% of the variation in W-15.

**Table 5:** Relationships of lamb weights on the 15th (W-15) and 45th (W-45) days with postnatal behaviours^†^ according to stepwise regression analyses

Model^‡^	Variables^§^	B	SE	β	VIF	*R* ^2^	RMSE	*P* -value	
W-15									
1						0.373	0.899	< 0.001	
	Constant	4.689	0.483						
	Grooming-30 min	2.269	0.404	0.611	1.000				
2						0.705	0.635	< 0.001	
	Constant	6.173	1.415						
	BW	1.063	0.159	0.584	1.301				
	Grooming-30 min	1.143	0.329	0.308	1.329				
	Stand-180 min	-1.430	0.354	-0.378	1.485				
	To udder	-1.000	0.460	-0.209	1.568				
W-45									
1						0.438	1.792	< 0.001	
	Constant	6.400	2.378						
	Grooming-30 min	4.405	0.834	0.574	1.074				
	Lying-150 min	4.412	1.477	0.442	1.993				
	Successful suck	-3.230	1.573	-0.311	2.081				
2						0.615	1.483	< 0.001	
	Constant	6.037	1.735						
	BW	2.133	0.372	0.568	1.297				
	Grooming-30 min	2.567	0.749	0.335	1.263				
	Stand-180 min	-1.936	0.689	-0.248	1.030				

^†^: Logarithmic transformations of postnatal behaviour data were used in regression analysis

^‡^: Model 1 shows the results of the best model determined as a result of the stepwise regression analysis applied with postnatal behaviours. Model 2 shows the results of the best model determined as a result of the stepwise regression analysis applied with postnatal behaviours and birth weight

^§^: BW= Birth weight; Grooming-30 min= Grooming by the dam during first 30 min; Stand-180 min= Stand duration during first 180 min; Lying-150 min= Lying duration during first 150 min

VIF: Variance inflation factor; R^2^: Coefficient of determination; RMSE: Root mean square error

**Table 6:** Relationships of lamb weight on the 60th day (W-60) and average daily gain (ADG) with postnatal behaviours^†^ according to stepwise regression analyses

Model^‡^	Variables^§^	B	SE	β	VIF	*R* ^2^	RMSE	*P* -value	
W-60									
1						0.301	2.558	< 0.001	
	Constant	11.071	1.374						
	Grooming-30 min	5.484	1.149	0.548	1.000				
2						0.529	2.139	< 0.001	
	Constant	9.044	2.502						
	BW	2.475	0.536	0.505	1.297				
	Grooming-30 min	3.284	1.080	0.328	1.263				
	Stand-180 min	-2.590	0.994	-0.254	1.030				
ADG									
1						0.425	33.925	< 0.001	
	Constant	-28.300	49.004						
	Grooming-30 min	79.651	15.684	0.555	1.059				
	Lying-150 min	87.145	26.224	0.467	1.752				
	Sucking-30 min	52.797	25.364	0.297	1.806				
2						0.492	32.229	< 0.001	
	Constant	-81.671	51.040						
	Grooming-30 min	61.398	16.529	0.428	1.303				
	Lying-150 min	84.566	24.934	0.453	1.754				
	BW	20.384	7.990	0.290	1.270				
	Sucking-30 min	47.636	24.181	0.268	1.818				

^†^: Logarithmic transformations of postnatal behaviour data were used in regression analysis

^‡^: Model 1 shows the results of the best model determined as a result of the stepwise regression analysis applied with postnatal behaviours. Model 2 shows the results of the best model determined as a result of the stepwise regression analysis applied with postnatal behaviours and birth weight

^§^: BW= Birth weight; Grooming-30 min= Grooming by the dam during first 30 min; Stand-180 min= Stand duration during first 180 min; Lying-150 min= Lying duration during first 150 min; Sucking-30 min= Sucking duration during first 30 min

VIF: Variance inflation factor; R^2^: Coefficient of determination; RMSE: Root mean square error

According to the results of the stepwise regression analyse for W-45, Model 1 included Grooming-30 min, lying duration during first 150 min (Lying-150 min) and latency to successful suck (Table 5). These variables explained 43.8% of variance in W-45 (*P* < 0.001). However, Model 2, which included birth weight, Grooming-30 min and Stand-180 min, explained 61.5% of the variance and was the best model for the prediction of W-45 (*P* < 0.001).

In terms of W-60, Grooming-30 min alone expressed 30.1% of the total variance (Model 1, *P* < 0.001; Table 6)). On the other hand, the best model for predicting W-60 included birth weight, Grooming-30 min and Stand-180 min (Model 2, *P* < 0.001). This model explained 52.9% of the variance.

Grooming-30 min, Lying-150 min and Sucking-30 min were the main determinants of ADG according to the results of Model 1 (*P* < 0.001). This model explained 42.5% of the variance in ADG. On the other hand, Model 2, which included Grooming-30 min, Lying-150 min, birth weight, and Sucking-30 min, was the best model for prediction of ADG, and this model explained 49.2% of the total variance.

## Discussion

### Effects of sex and birth type of lamb and parity of dam on early postnatal behaviours and lamb growth

One of the secondary objectives of this study is to determine whether the sex and birth type of the lamb and the parity of the dam influence early postnatal lamb behaviours. Lamb sex and birth type had no significant influence on latencies of postnatal lamb behaviours such as shakes head, to knees, attempt to stand, stands, to udder, suck attempt, and successful suck in Kivircik sheep. These results are partially consistent with many previous reports (Abdul-Rahman and Yaro 2010; Cloete et al. 2002; Dwyer 2003). Abdul-Rahman and Yaro (2010) stated that the fact that single born lambs are generally heavier than their twin counterparts causes the single lambs to be more vigorous, enabling them to stand up earlier. In the current study, although single lambs successfully sucked numerically earlier than twins (42 min vs. 56 min), the effect of birth type on the latency of successful suck was not significant (*P* = 0.111). Also, there was no significant difference between single and twin born lambs in terms of suckling duration during the first 180 min. These results may indicate that although the birth weight of twin lambs was lower than that of single lambs, it did not pose a problem regarding the vigour required for the successful sucking of twin Kivircik lambs.

In the current study, lambs of fourth parity ewes sucked earlier than that of second ones, whereas lambs of primiparous ewes tended to suck later than those of multiparous dams. This observed trend is biologically plausible, as maternal experience and instinct are known to strengthen with increasing parity. Multiparous ewes generally show stronger maternal responsiveness, more effective stimulation and guidance of the lamb, and fewer non-cooperative behaviours during early suckling attempts (Dwyer and Lawrence 1998; Nowak and Poindron 2006). These behaviours facilitate rapid udder seeking by lambs and shorten the latency to successful suckling. In contrast, primiparous ewes often exhibit lower maternal competence and a higher frequency of non-cooperative behaviours, which may delay the lamb’s progress towards the udder and prolong the latency to successful suck (Dwyer 2003). Consistent with these findings, Karaca et al. (2023) reported that lambs of multiparous Norduz ewes started to suck in a shorter time than those of lambs of biparous and primiparous ones, owing to the maternal facilitation of the process. In another previous study (Dwyer 2003), seeks udder, suck attempt, and successful suck behaviours were significantly slower in lambs born to first parity ewes compared to that of second, third and fourth parity ewes. Dwyer (2003) suggests that parity affects lamb behaviour due to physiological and neuroendocrine differences between first-time and experienced mothers. In addition to behavioural factors, udder morphology, including symmetry, length and thickness of the teats, and teat placement, can also influence the ease of suckling and may contribute to unexplained variation in sucking latency (Devi et al. 2022). However, udder conformation traits were not recorded in the present study, and therefore possible parity-related differences in udder morphology could not be assessed.

In the current study durations of sucking, standing, and lying were not influenced by lamb sex, birth type and parity of dam. These results indicate that the variation attributable to these factors was either minimal or insufficient to reach statistical significance within the conditions of this study. Consistent with the present findings, Simitzis et al. (2016) reported no significant differences among singleton, first twin, second twin lambs regarding time standing during the first hour after birth. Karaca et al. (2023) also observed similar total sucking durations during the 3-h postnatal period in Norduz lambs born to primiparous, biparous, and multiparous ewes. In the current study, the lack of statistically significant differences may be attributed to limited biological variability in these behaviours (Dwyer and Lawrence 1998), or potentially to insufficient statistical power to detect subtle effects. However, the consistency with results of previous studies supports the interpretation that early postnatal sucking and locomotor behaviours are relatively stable traits and may be less sensitive to demographic factors such as sex, litter size, or parity (Dwyer 2003; Nowak and Poindron 2006).

As expected, the duration of grooming by the dam was lower in twin lambs than in single lambs. Although ewes from prolific breeds typically exhibit an increase in total grooming activity following the birth of twin lambs (Dwyer and Lawrence 1998; O’Connor et al. 1992), this increase is necessarily distributed between two neonates. Consequently, the amount of grooming each twin receives is often reduced simply because the ewe must divide a limited amount of time and attention between both lambs during the critical early postnatal period. In indigenous sheep breeds such as the Kivircik, where prolificacy is generally lower and strong behavioural adaptations to twin rearing may be less pronounced, ewes giving birth to twins may not increase the grooming time sufficiently, and therefore twin-born lambs may receive less grooming attention than single lambs (Ekiz et al. 2007). Supporting this view, Simitzis et al. (2016) reported that second-born twins in Karagouniko, Chios, and Orini Epirou dairy breeds received less grooming than singles or first-born twins, underscoring the challenges ewes face in dividing maternal attention among multiple offspring.

In the present study, the sex of the lamb and the parity of the dam had no influence on the weights of lambs at 15, 45, and 60 days and the average daily gain. The lack of significant effects may indicate that, under the management and nutritional conditions of this flock, potential mechanisms such as sex-related differences in metabolic demands or parity-related variation in maternal milk yield (Ghafouri-Kesbi and Notter 2016; Pesántez-Pacheco et al. 2019) were not strong enough to produce significant differences in early growth. Moreover, the absence of sexual dimorphism in pre-weaning growth is not unexpected, as sex-related divergence in growth performance typically becomes more evident after weaning, when hormonal influences on tissue accretion increase (Ozcan et al. 2004).

Single lambs were heavier than twins at all ages studied. A decreased growth rate in twin lambs compared with single lambs was expected due to reduced milk availability and diminished protein and energy intake as they must share the mother’s milk with their twin sibling. These results are also consistent with previous studies on Kivircik lambs (Ekiz and Altinel 2006; Ekiz et al. 2016), which documented a higher growth rate in single-born lambs than in their twin counterparts.

### Prediction of lamb growth using behaviours in the first three hours postnatal

One of the primary goals of this study is to determine whether there is a relationship between early postnatal lamb behaviours and lamb growth rate and to develop the regression model that most accurately predicts the growth rate using early postnatal behaviours and birth weight in lambs. Previous studies have indicated that maternal care and the postnatal sucking behaviour of neonates influenced lambs’ growth, health, and welfare. The coordinated expression of appropriate behaviours by both the ewe and the lamb plays an important role in ensuring the healthy growth of lambs (Dwyer 2003; Han et al. 2023).

In the current study, two different models were considered using the stepwise regression procedure to examine the relationship between postnatal lamb behaviours and lamb growth rate. The poorest prediction (lowest R^2^ and highest RMSE value) was obtained using a model that included only behaviours in the postnatal 3 h (Model 1). With Model 1, coefficients of determination ranging from 0.301 to 0.438 were obtained for lamb growth characteristics. The model incorporating postnatal behaviours along with lamb birth weight (Model 2) provided the best prediction with R^2^ values ranged from 0.492 to 0.705. These findings indicate that using only behaviours for predicting early-stage lamb growth results in poor to moderate predictive ability. However, when birth weight is added to the model, there is an important improvement in the model’s explanatory power. With Model 2, it is possible to predict the early lamb growth rate with moderate accuracy. Likewise, Mialon et al. (2023) observed that lamb birth weight served as an additional predictor of growth rate, alongside early-life behavioural indicators.

Although there have been previous studies regarding predicting pre-weaning survival using postnatal ewe and lamb behaviours (Cloete et al. 2021; Mialon et al. 2023; Odevci et al. 2021), research specifically targeting the prediction of lamb growth rate using postnatal lamb behaviours as predictors is quite limited. Hence, comparing the coefficient of determination values obtained for regression models in the current study with those of previous similar studies is not feasible. In a previous study (Mialon et al. 2023) conducted partially with a similar aim to ours, the relationship between the following predictors and lamb growth rate was examined: body condition score of the dam, litter size, lamb sex, birth weight, rectal temperature, handling score, vigour score, and sucking score. They observed that the model including vigour score, sucking score, birth weight, and sex was the best model explaining average daily gain with an R^2^ value of 0.212. The R^2^ values determined for ADG in two models in the current study are higher than those reported by Mialon et al. (2023). Although Darwish et al. (2010) did not examine the relationship between postnatal lamb behaviours and pre-weaning growth performance, they reported that higher growth rates and weaning weights of Crossbred Finn lambs compared to purebred Rahmani lambs may partially attributed to the higher activity levels exhibited by these lambs at birth. Çakmakçı et al. (2021) applied an open field test to 30-day-old Karakaş breed lambs to evaluate their behavioural responses to novelty and isolation and divided the lambs into two groups: proactive and reactive. Researchers observed that proactive and reactive lambs had similar weights at days 45 and 60, but they determined that from day 75 until the end of the study (day 120), the growth rate of reactive lambs was higher.

In fact, numerous studies have been conducted previously to predict lamb weights or ADG at various periods (Ibrahim et al. 2021; Iqbal et al. 2019; Sun et al. 2020). These studies typically utilise various body measurements as predictors, aiming to predict body weights at the age at which measurements are taken. The R^2^ values around 0.60–0.90 are commonly reported in these studies. The current study differs from these studies by using data collected during the three-hour postnatal period as predictors instead of parameters measured at weighing time. Therefore, obtaining a lower R^2^ value compared to studies using body measurements as predictors is an expected outcome. On the other hand, in a study where milk yield and milk composition of the dam were used as predictors for weaning weight, the R^2^ values (between 0.04 and 0.21) were much lower than those of the current results (Shihab 2024).

When examining which behavioural characteristics are included in Models 1 and 2, it is observed that “grooming by the dam during the first 30 minutes” is included in the regression equations established for all growth traits. In all equations, it is observed that the regression coefficients associated with Grooming-30 min are positive. These results indicate that as postnatal grooming time by the dam during the first 30 minutes’ increases, the body weights of lambs at various periods also increase. Grooming by the dam dries, cleans and stimulates the lamb, and it also facilitates the formation of a strong bond with the newborn lamb through recognition of its odour (Nowak and Poindron 2006). Everett-Hincks et al. (2005) reported that lambs by ewes that stayed close to their litter during tagging and did not return to the flock grew faster. On the other hand, Yilmaz et al. (2011) found no significant relationship between maternal behaviours and lamb growth.

In the current study, Stand-180 min was included in the regression equations established for W-15, W-45, and W-60 with Model 2. Lying-150 min was included in Model 1 for W-45 and in Models 1 and 2 for ADG. The regression coefficient for Stand-180 min was negative, while it was positive for Lying-150 min. Everett-Hincks et al. (2005) similarly reported that lambs which took longer to stand after tagging exhibited faster growth until weaning, and that each additional minute spent lying was associated with an extra 10 g/day of growth. A plausible explanation is that lying down reduces maintenance energy expenditure compared to standing, thereby allowing more energy to be diverted to tissue accretion. Indeed, studies in other species confirm that standing requires more energy than lying down, suggesting that increased resting time conserves energy for growth (Ortigues et al. 1994).

The latency times to udder and successful suck were also significant determinants of W-15 and W-45, respectively. It has been determined that lambs exhibiting these behaviours earlier would have higher body weights. This association is biologically plausible, as early suckling ensures the prompt intake of maternal colostrum. Colostrum is crucial as the primary source of energy and passive immunoglobulins for newborn lambs, given their inability to acquire antibodies in utero (Yang et al. 2025). Supporting to the current results, Madani et al. (2013) observed that lambs initiating suckling shortly after birth exhibited greater weight gain compared to those displaying slower behavioural progress after birth.

Sucking-30 min is observed to be one of the significant predictors of ADG. It is anticipated that as the sucking duration during the first 30 minutes’ postnatal increases, ADG will also increase. Mialon et al. (2023) reported that lambs that showed weak sucking activity had a lower growth rate. Low postnatal sucking activity can be attributed to various factors, including physiological or fitness-related issues. Some lambs may inherently possess a weak sucking reflex, which typically develops in utero (Mialon et al. 2023). Additionally, diminished sucking activity might indicate depleted body reserves, insufficient post-birth nursing by the dam during the initial hours, or low-quality colostrum (Dwyer et al. 2016; Mialon et al. 2023).

### Limitations of the study

Although the sample size limits the statistical power to detect small differences in growth traits, the study still provides valuable insights into the relationship between early postnatal behaviours and lamb growth under controlled management conditions in Kivircik lambs. While these early behaviours showed moderate associations with growth up to 60 days of age, the findings should be interpreted with caution, as growth performance is also influenced by factors such as maternal nutrition, milk yield and quality, the lambs’ diet, and environmental conditions. One of the limitations is that udder conformation traits were not recorded in the study, which prevented assessment of whether parity-related differences in udder morphology contributed to variation in sucking latency.

## Conclusion

The study concluded that the growth rates of Kivircik lambs over the first 60 days postnatal could be predicted with moderate accuracy (with R^2^ values ranging from 0.492 to 0.705) by considering birth weight alongside lamb behaviours observed during the initial 3 h postnatal as explanatory variables. “Grooming by the dam during the first 30 min” and “standing duration during the first 180 min” emerge as the most important behavioural predictors of lamb growth. In addition, regression models developed for growth traits at various periods included Lying-150 min and Sucking-30 min, along with latency times to udder and successful suck as potential indicators of lamb growth. Another conclusion of the study is that as the growth period progresses, the prediction accuracy decreases.

## Data Availability

The datasets of the current study are available from the corresponding author on reasonable request.

## References

[CR1] Abdul-Rahman II, Yaro M (2010) The effect of sex, birth weight and type of birth on neonatal behaviour of Djallonke sheep and West African Dwarf goats. Glob Vet 4(4):409–415

[CR2] Anonymous (2011) Regulation on the welfare and protection of animals used for experimental and other scientific purposes. Turkish Official Gazette (No: 28141) 13 December 2011

[CR3] Bruce M, Young JM, Masters DG, Refshauge G, Thompson AN, Kenyon PR, Behrendt R, Lockwood A, Miller DW, Jacobson C (2021) The impact of lamb and ewe mortality associated with dystocia on Australian and New Zealand sheep farms: A systematic review, meta-analysis and bio-economic model. Pre Vet Med 196:105478. 10.1016/j.prevetmed.2021.105478 10.1016/j.prevetmed.2021.105478 34487918

[CR4] Çakmakçı C, Karaca S, María GA (2021) Does coping style affect behavioral responses and growth performance of lambs weaned at different ages? J Vet Behav 42:64–74. 10.1016/j.jveb.2020.10.009

[CR5] Cloete SWP, Scholtz AJ, Gilmour AR, Olivier JJ (2002) Genetic and environmental effects on lambing and neonatal behaviour of Dormer and South African Mutton Merino lambs. Livest Prod Sci 78:183–193. 10.1016/S0301-6226(02)00117-3

[CR6] Cloete SWP, Burger M, Scholtz AJ, Cloete JJE, Nel CL, Gilmour AR, Van Wyk JB (2021) The genetics of perinatal behaviour of merinos in relation to lamb survival and lambs weaned per ewe mated. Appl Anim Behav Sci 236:105217. 10.1016/j.applanim.2021.105217

[CR7] Darwish RA, Abou-Imail UA, El-Kholya SZ (2010) Differences in post-parturient behaviour, lamb performance and survival rate between purebred Egyptian Rahmani and its crossbred Finnish ewes. Small Rumin Res 89:57–61. 10.1016/j.smallrumres.2009.12.003

[CR8] Devi I, Mallick PK, Mohapatra A, Shinde AK, Kumar A (2022) Effect of udder morphology on milk yield and suckling behaviour of patanwadi lambs. Indian J Small Rumin 28:96–100. 10.5958/0973-9718.2022.00011.3

[CR10] Dwyer CM (2003) Behavioural development in the neonatal lamb: effect of maternal birth related factors. Theriogenology 59:1027–1050. 10.1016/S0093-691X(02)01137-8 12517402 10.1016/s0093-691x(02)01137-8

[CR12] Dwyer CM (2008) Individual variation in the expression of maternal behavior: A review of the neuroendocrine mechanisms in the sheep (Ovis aries). J Neuroendocrinol 220:526–535. 10.1111/j.1365-2826.2008.01657.x 10.1111/j.1365-2826.2008.01657.x 18266950

[CR9] Dwyer CM, Lawrence AB (1998) Variability in the expression of maternal behavior in primiparous sheep: effects of genotype and litter size. Appl Anim Behav Sci 58:311–330. 10.1016/S0168-1591(97)00148-2

[CR14] Dwyer CM, Lawrence AB (1999) Does the behaviour of the neonate influence the expression of maternal behaviour in sheep? Behaviour 136(3):367–389

[CR11] Dwyer CM, Calvert SK, Farish M, Donbavand J, Pickup HE (2005) Breed, litter and parity effects on placental weight and placentome number, and consequences for the neonatal behavior of the lamb. Theriogenology 63:1092–1110. 10.1016/j.theriogenology.2004.06.003 15710196 10.1016/j.theriogenology.2004.06.003

[CR13] Dwyer CM, Conington J, Corbiere F, Holmøy IH, Muri K, Nowak R, Gautier JM (2016) Improving neonatal survival in small ruminants: science into practice. Animal 10(3):449–459. 10.1017/S1751731115001974 26434788 10.1017/S1751731115001974

[CR15] Ekiz B, Altinel A (2006) The growth and survival characteristics of lambs produced by crossbreeding Kivircik ewes with F2 rams with the German Black-Headed Mutton genotype. Turk J Vet Anim Sci 30:507–512

[CR17] Ekiz B, Kocak O, Ozcan M, Yilmaz A (2007) Effects of parity and litter size on maternal behaviour in Kivircik ewes. Acta Agric Scand Anim Sci 57(2):81–88. 10.1080/09064700701766726

[CR16] Ekiz B, Kocak O, Yalcintan H, Yilmaz A (2016) Effects of suckling duration on growth, slaughtering and carcass quality characteristics of Kivircik lambs. Trop Anim Health Prod 48:395–401. 10.1007/s11250-015-0964-7 26676241 10.1007/s11250-015-0964-7

[CR18] Ekiz B, Yalcintan H, Kocak O, Kecici PD (2024) Prediction of lamb survival using machine learning algorithms with neonatal lamb behaviors and maternal behavior score in Kivircik lambs. J Vet Beh 74:37–45. 10.1016/j.jveb.2024.06.008

[CR19] European Union (2010) European Union Directive 2010/63/EU of the European parliament and of the council of 22 September 2010 on the protection of animals used for scientific purposes. Official J Eur Union L 276:33–79

[CR20] Everett-Hincks JM, Lopez-Villalobos N, Blair HT, Stafford KJ (2005) The effect of maternal behavior score on lamb and litter survival. Livest Prod Sci 93:51–61. 10.1016/j.livprodsci.2005.05.006

[CR21] Eyduran E (2016) The possibility of using data mining algorithms in prediction of live body weights of small ruminants. Can J Appl Sci 1:1–4

[CR22] Fonseca VFC, Morais LKC, Saraiva EP, de Sousa WH, Filho ECP, dos Santos JDC, Neta GCX, Ungerfeld R, Freitas-de-Melo A (2024) Ewe-lamb bond at birth and during lactation in an equatorial semi-arid environment is better in a native than in an introduced breed. Appl Anim Beh Sci 278:106362. 10.1016/j.applanim.2024.106362

[CR23] Ghafouri-Kesbi F, Notter DR (2016) Sex influence on genetic expressions of early growth in Afshari lambs. Arch Anim Breed 59:9–17. 10.5194/aab-59-9-2016

[CR24] Han C, Wang Y, Li F, Wang Z, Yang Y, Lv S, Wang H (2023) Effects of lamb sex and ewe parity on suckling-related neonatal behaviors and weaning weight of small-tailed han lambs. J Vet Behav 59:36–45. 10.1016/j.jveb.2022.11.001

[CR25] Ibrahim A, Artama WT, Budisatria IS, Yuniawan R, Atmoko BA, Widayanti R (2021) Regression model analysis for prediction of body weight from body measurements in female Batur sheep of Banjarnegara District, Indonesia. Biodiversitas 22:2723–2730. 10.13057/biodiv/d220721

[CR26] Iqbal F, Ali M, Huma ZE, Raziq A (2019) Predicting live body weight of Harnai sheep through penalized regression models. J Anim Plant Sci 29(6):1541–1548

[CR27] Karaca S, Aydoğdu N, Ser G (2023) Effect of maternal experience and body condition on patterns of ewe-lamb bonding behaviors and pre-weaning growth performance of lambs. J Vet Behav 67:1–7. 10.1016/j.jveb.2023.07.003

[CR28] Khan MA, Ahmad M, Popp J, Oliah J (2020) US policy uncertainty and stock market nexus revisited through dynamic ARDL simulation and threshold modelling. Mathematics 8:2073. 10.3390/math8112073

[CR29] Kilkenny C, Browne WJ, Cuthill IC, Emerson M, Altman DG (2010) Improving bioscience research reporting: The ARRIVE guidelines for reporting animal research. PLOS Biol 8(6):e1000412. 10.1371/journal.pbio.1000412 20613859 10.1371/journal.pbio.1000412 PMC2893951

[CR30] Madani T, Allouche L, Saffidine N, Kaouane N, Belkasmi F, Semara L (2013) Maternal and neonatal behaviors of Ouled Djellal sheep breed and their effects on production parameters. Small Rumin Res 114:46–50. 10.1016/j.smallrumres.2013.06.003

[CR31] Mialon MM, Nowak R, Falourd P, Marcon D, Lardy R, Boivin X (2023) Are early-life lambs’ characteristics and behavioural reactivity related to later survival and growth performance during artificial feeding? Appl Anim Behav Sci 262:105918. 10.1016/j.applanim.2023.105918

[CR33] Nowak R (1996) Neonatal survival: contributions from behavioural studies in sheep. Appl Anim Behav Sci 49:61–72. 10.1016/0168-1591(95)00668-0

[CR32] Nowak R, Poindron P (2006) From birth to colostrums: early steps leading to lamb survival. Reprod Nutr Dev 46:431–446. 10.1051/rnd:2006023 16824451 10.1051/rnd:2006023

[CR34] O’Connor CE, Lawrence AB, Wood-Gush DGM (1992) Influence of litter size and parity on maternal behaviour at parturition in Scottish Blackface sheep. Appl Anim Behav Sci 33:345–355. 10.1016/S0168-1591(05)80071-1

[CR35] Odevci BB, Emsen E, Aydin MN (2021) Machine learning algorithms for lamb survival. Comput Electron Agric 182:105995. 10.1016/j.compag.2021.105995

[CR36] Ortigues I, Martin C, Vermorel M, Anglaret Y (1994) Energy cost of standing and circadian changes in energy expenditure in the preruminant calf. J Anim Sci 72:2131–2140 7982844 10.2527/1994.7282131x

[CR37] Ozcan M, Ekiz B, Yilmaz A, Ceyhan A (2004) The effects of some environmental factors affecting on the growth and greasy fleece yield at first shearing of Turkish Merino (Karacabey Merino) lambs. İstanbul Üniv. Vet Fak Derg 30:159–167

[CR38] Pesántez-Pacheco JL, Heras-Molina A, Torres-Rovira L, Sanz-Fernández MV, García-Contreras C, Vázquez-Gómez M, Feyjoo P, Cáceres E, Frías-Mateo M, Hernández F, Martínez-Ros P, González-Martin JV, González-Bulnes A, Astiz S (2019) Influence of maternal factors (weight, body condition, parity, and pregnancy rank) on plasma metabolites of dairy ewes and their lambs. Animals 9(4):122. 10.3390/ani9040122 30925737 10.3390/ani9040122 PMC6523727

[CR39] Rooke JA, Arnott G, Dwyer CM, Rutherford KMD (2017) Impact of maternal stress and nutrition on behavioural and physiological outcomes in young lambs. Anim Welf 26:403–415

[CR40] Shihab OH (2024) Description of growth curve by non-linear function and prediction of weaning weight from milk production and composition in Turkish Awassi sheep. Int J Vet Sci Anim Husb 9(1):1300–1303

[CR41] Simitzis P, Galani K, Vasiliou P, Koutsouli P, Bizelis I (2016) Effect of breed and litter size on the display of maternal perinatal and offspring postnatal behavior in dairy sheep. J Vet Behav 13:10–18. 10.1016/j.jveb.2016.02.008

[CR42] Sun MA, Hossain MA, Rahman MM, Hossain MM, Hashem MA (2020) Different body measurement and body weight prediction of jamuna basin sheep in Bangladesh. SAARC J Agric 18(1):183–196. 10.3329/sja.v18i1.48392

[CR43] Yang C, Du M, Ahmad AA, Cheng Y, Gebeyew K (2025) Passive immunity establishment through colostral igg absorption in neonatal ruminants: foundation for efficient ruminant production. Animals 15:3093. 10.3390/ani15213093 41227424 10.3390/ani15213093 PMC12609626

[CR44] Yilmaz A, Karaca S, Bingöl M, Kor A, Kaki B (2011) Effects of the maternal behavior score (MBS) on weaning weight and litter survival in sheep. Front Agric Food Technol 7(4):1–5

